# Pevonedistat Suppresses Pancreatic Cancer Growth *via* Inactivation of the Neddylation Pathway

**DOI:** 10.3389/fonc.2022.822039

**Published:** 2022-01-26

**Authors:** Junfeng Xu, Zheng Li, Qifeng Zhuo, Zeng Ye, Guixiong Fan, Heli Gao, Shunrong Ji, Xianjun Yu, Xiaowu Xu, Wensheng Liu, Wenyan Xu

**Affiliations:** ^1^ Department of Pancreatic Surgery, Fudan University Shanghai Cancer Center, Shanghai, China; ^2^ Department of Oncology, Shanghai Medical College, Fudan University, Shanghai, China; ^3^ Shanghai Pancreatic Cancer Institute, Shanghai, China; ^4^ Pancreatic Cancer Institute, Fudan University, Shanghai, China

**Keywords:** neddylation pathway, pevonedistat, pancreatic cancer therapy, cell growth, cell cycle

## Abstract

**Background:**

The neddylation pathway is aberrantly overactivated in multiple human cancers and has been indicated as an effective target for anticancer therapy in clinical trials. We aimed to study whether the neddylation pathway is upregulated in pancreatic cancer and whether pevonedistat, a first-in-class anticancer agent specifically targeting this pathway, will suppress cancer tumorigenesis and progression.

**Methods:**

We evaluated the expression pattern of neddylation pathway components in 179 pancreatic adenocarcinoma (PAAD) compared with 171 normal tissues from The Cancer Genome Atlas (TCGA) dataset and further assessed PAAD patient prognosis with high neddylation pathway expression *via* Gene Expression Profiling Interactive Analysis (GEPIA). We then analyzed malignant cancer phenotypes both *in vitro* and *in vivo*, as well as intrinsic molecular mechanisms upon pevonedistat treatment.

**Results:**

We found that the neddylation pathway was hyperactivated in pancreatic cancer. Patients with high neddylation pathway expression exhibited worse prognoses. Pevonedistat significantly inhibited the cancer cell cycle, cell growth, and proliferation; increased cell apoptosis; and decreased cancer cell xenografts in a mouse model. Mechanistically, pevonedistat treatment and the siRNA knockdown neddylation pathway were able to remarkably induce the accumulation of Wee1, p27, and p21. Further mechanistic studies revealed that pevonedistat mainly impaired the ubiquitination level and delayed the protein degradation of Wee1, p27, and p21.

**Conclusions:**

Our results showed that pevonedistat targeted the overexpression of the neddylation pathway in pancreatic cancer to induce cell growth suppression by inducing the accumulation of the cell cycle regulators Wee1, p27, and p21, which provides sound evidence for the clinical trial of pevonedistat for pancreatic cancer therapy.

## Background

Pancreatic cancer is one of the most malignant cancers and the fourth leading cause of cancer death worldwide (1, 2). The 5-year survival rate for localized pancreatic cancer is only 39%; for the regional stage, it is 13%; for the distant stage, it is 3%; and for all stages combined, it is 10% (1). During the past decades, the three major treatments for pancreatic cancer, including surgery, chemotherapy, and radiation, have been widely applied in clinical therapy (2, 3). Despite improvements in recent therapeutic strategies and approaches (4, 5), little improvement has been achieved in the survival rate in pancreatic cancer because of drug insensitiveness and tolerance, drug resistance after treatment, severe adverse effects upon treatment, and relatively low efficacy for pancreatic cancer therapy (6), resulting in a high rate of local recurrences, an increased risk of distant metastases, and poor prognosis in pancreatic cancer patients (7). Individuals even have similar clinical features at diagnosis but acquire quite heterogeneous prognoses (8). Independent and personalized anticancer targets are urgently needed to improve the anticancer efficacy of pancreatic cancer and allow better-guided clinical decision-making.

The neddylation pathway, a post-translational modification process, includes a three-step enzymatic cascade: NEDD8-activating enzyme E1 (NAE, a heterodimer of 2 subunits NAE1 and UBA3) activates the ubiquitin-like small molecular precursor NEDD8 (neural precursor cell expressed, developmentally downregulated 8), NEDD8-conjugating enzyme E2 M (UBC12) then transfers NEDD8 to E3 ubiquitin ligases, and E3 ligases catalyze the covalent binding of NEDD8 to downstream client substrates (9, 10). Cullin family proteins are the best-known substrates of the neddylation pathway and consist of Cullins 1, 2, 3, 4a, 4b, 5, and 7 (11). As an essential part of Cullin-RING E3 ubiquitin ligase (CRL), once neddylated, it will be activated by conformational changes to perform biological functions that trigger targeted protein ubiquitination and subsequent degradation (12, 13) and further regulate numerous biological processes, such as gene transcription, protein translation, cell division, cell cycle, signal transduction, and immune responses (14, 15), whereas dysfunction of the neddylation pathway leads to tumorigenesis and cancer progression due to disruption of Cullin neddylation (16, 17). Recent studies reported that neddylation pathway members, such as NAE1, UBC12, and NEDD8, were aberrantly overexpressed in several human cancers with a poor prognosis (18–20), indicating that the neddylation pathway is a valid anticancer target.

Pevonedistat, a specific small inhibitor of NAE, is currently applied in several phase I/II clinical trials for myelodysplastic syndromes, malignant solid neoplasms, acute myeloid leukemia, and acute myelogenous leukemia (http://www.clinicaltrials.gov). It has been reported as having anticancer activity in multiple cancer xenograft models by inhibiting the neddylation pathway and therefore abolishing Cullin neddylation and CRL activities that induce the accumulation of tumor-suppressive substrates to impair cancer cell growth and result in cell apoptosis and senescence (9, 19, 21–26). Recent preclinical studies have shown pevonedistat to be an effective therapy drug combined with chemo/radiotherapy or a single anticancer agent with well-tolerated toxicity among different types of cancers (27–29).

There have been a few small studies evaluating the association of the neddylation pathway with pancreatic cancer, as well as the anticancer efficacy of pevonedistat in pancreatic cancer, and the results were similar. Langdon et al. (30) found that the combination of the bromodomain inhibitor JQ1 and pevonedistat can prevent *in vivo* xenograft growth of pancreatic cancer cells by altering reactive oxygen species (ROS) production and leading to DNA damage response (DDR) defects. Other studies (31, 32) reported that pevonedistat cooperated with gemcitabine or cisplatin to treat pancreatic cancer, at least in part, by increasing chemosensitization to chemotherapy agents by accumulating proapoptotic proteins, such as NOXA and ERBIN. Wei et al. (25) consistently showed that pevonedistat sensitized pancreatic cancer cells to radiotherapy *via* CDT1- and Wee1-induced DNA damage and cell cycle arrest. These findings have demonstrated pevonedistat as a potential clinical adjunctive strategy for the treatment of pancreatic cancer (33). Nevertheless, the underlying mechanisms of the anti-pancreatic cancer effects of pevonedistat remain elusive. In the current study, the anti-pancreatic cancer efficacy and intrinsic molecular mechanisms of pevonedistat function were intensively defined. We found that neddylation suppression by pevonedistat alone significantly inhibited pancreatic cancer malignant phenotypes by inducing G2 phase cell cycle arrest and Wee1/p27/p21 axis-directing apoptosis. Our study provides solid evidence that the neddylation pathway is overactivated in pancreatic cancer and indicates pevonedistat anti-pancreatic cancer efficacy.

## Materials and Methods

### Cell Culture and Reagents

Human pancreatic cancer cell lines Miapaca-2, Capan-1, SW1990, CFPAC-1, and Capan-2 were obtained from the American Type Culture Collection. Cells were routinely cultured in Dulbecco’s modified Eagle’s medium or Roswell Park Memorial Institute (RPMI) 1640 medium (Gibco, Thermo Fisher Scientific, Grand Island, NY, USA) with 10% fetal bovine serum (FBS) (Biochrom, Holliston, MA, USA) and 1% penicillin-streptomycin solution, a humidified atmosphere of 5% CO_2_, and 95% air at 37°C. Pevonedistat was purchased from MCE and used for *in vitro* studies as described in the manufacturer’s instruction.

### Collection of Pancreatic Cancer Tissue Specimens

Human pancreatic cancer tissues and paired normal tissues used in this study were the same as in our previous study, which was histopathologically and clinically diagnosed at Fudan University Shanghai Cancer Center from 2010 to 2011, and prior patient informed consent and approval from the Institutional Research Ethics Committee were obtained (34). Anti-NAE1 (Invitrogen), anti-Ubc12 (ABclonal), and anti-Rbx1 (ABclonal) were used to detect protein expression.

### Cell Proliferation and Clonogenic Survival Assays

For the cell proliferation assay, cells were seeded into 96-well plates at 2,500 cells per well in quadruplicate and cultured for 72 h. Cell viability was determined with the Cell Counting Kit-8 kit (Beyotime) according to the manufacturer’s instructions. For the clonogenic assay, cells were seeded into 12-well plates (150 cells per well) in triplicate and cultured for 10 days. Then, colonies were fixed, stained with crystal violet, and counted under an inverted microscope (Olympus). Representative results of three independent experiments with similar trends are presented.

### Immunoblotting and Cycloheximide-Chase Analysis

Cell lysates were prepared for immunoblotting (IB) analysis using antibodies against Ubc12, Cullin 1, Rbx1 (ABclonal), p27, Wee1, p21 (Cell Signaling), and NAE1 (Invitrogen). β-Actin (ABclonal) was used as the loading control. For cycloheximide (CHX)-chase experiments, cells were pretreated with 1.0 μM of pevonedistat for 24 h, further treated with 50 μg/ml of CHX (Sigma) with pevonedistat or dimethyl sulfoxide (DMSO) for the indicated time points, and subjected to IB using antibodies against Wee1, p27, and p21 with actin as a loading control.

### *In Vivo* Ubiquitination Assay

To detect endogenous Wee1/p27/p21 ubiquitination, 1 × 10^6^ Miapaca-2 and Capan-1 cells were treated with pevonedistat vs. DMSO for 24 h and further treated with MG-132 for more than 24 h. After that, cells were harvested and lysed in NP-40 lysis buffer (Beyotime, catalog: P0013F) under denaturing conditions. After centrifugation at 4°C, cell lysates were incubated with 5 μl of anti-Wee1/p27/p21 Ab or rabbit IgG control antibody at 4°C overnight. Ten microliters of Protein A+G agarose (Santa Cruz Biotechnology) was then added to the cell supernatant for incubation at 4°C for 1 h. Following washing 6 times with NP-40 lysis buffer, the beads were subjected to IB with anti-ubiquitin Ab (Cell Signaling).

### Propidium Iodide Staining and Fluorescence-Activated Cell-Sorting Analysis

Cell cycle profile was determined by propidium iodide (PI) staining and fluorescence-activated cell sorting (FACS) analysis as described previously (20). Cells were harvested and fixed in 70% ethanol at −20°C overnight, stained with PI (36 μg/ml; Sigma) containing RNase (10 μg/ml; Sigma) at 37°C for 30 min, and then analyzed for a cell cycle profile by CyAn ADP (Beckman Coulter). Data were analyzed with ModFit LT software (Verity Software House).

### Detection of Apoptosis

Cells were transfected with 100 nM of siRNA oligonucleotides or treated with the indicated concentration of pevonedistat for 72 h and then harvested for Annexin V-FITC/PI staining. Apoptosis was determined with the Annexin V-FITC/PI Apoptosis Kit (Beyotime) according to the manufacturer’s instructions.

### RNA Interference

Miapaca-2 or Capan-1 cells were transfected with siRNA oligonucleotides using Lipofectamine 3000 reagent according to the manufacturer’s instructions. The sequence of siRNAs are as follows: for NEDD8: siNEDD8-1#: 5′-GAUUGAGAUUGACAUUGAACCTT-3′; siNEDD8-2#: 5′-GAUGAAUGAUGAGAAGACAGCTT-3′; for NAE1: siNAE1: 5′-GGGUUGUGCUUUAGUCUGUTT-3′; for UBC12: siUBC12-1#: 5′-GGGCUUCUACAAGAGUGGGAAGUTT-3′; siUBC12-2#: 5′-CGAUAAACUCCAUAAUUUAUGTT-3′; for RBX1: siRBX1-1#: 5′-GACUUUCCCUGCUGUUACCUAATT-3′; siRBX1-2#: 5′-GCUGUUACCUAAUUACAAAUUTT-3′; for control scrambled siRNA, siControl: 5′-UUCUCCGAACGUGUCACGUTT-3′. All the above siRNAs were purchased from GenePharma (Shanghai, China).

### Subcutaneous-Transplantation Tumor Model

For the tumor formation assay, 4-week-old female athymic nude mice were purchased from the Shanghai Experimental Animal Center (Shanghai, China). A total of 1 × 10^7^ stable cells were subcutaneously injected into the front armpit of nude mice. The next week, the mice harboring xenograft tumors were randomly divided into two groups (n = 6 per group) and treated with 10% 2-hydroxypropyl-β-cyclodextrin (HPBCD) or pevonedistat (20 mg/kg, s.c.) twice a day on a 3-day-on/2-day-off schedule for four cycles. Tumor size was measured by a Vernier caliper and calculated as (length × width2)/2. Mice were sacrificed at the end of the study by being placed in a carbon dioxide chamber. All procedures were performed in accordance with the National Institutes of Health Guide for the Care and Use of Laboratory Animals.

### Bioinformatics Analysis

The Cancer Genome Atlas (TCGA) RNA-Seq and corresponding clinical data were based upon data generated by TCGA Research Network (http://gepia.cancer-pku.cn/). RNA-Seq analysis was performed in Gene Expression Profiling Interactive Analysis (GEPIA) for the data from 179 pancreatic cancer tissues and 171 adjacent normal tissues.

### Statistical Analysis

All data were presented as mean ± SEM. Student’s t-test was used for the comparison of parameters between two groups, and the statistical significance of differences between groups was assessed using GraphPad Prism5 software. Three levels of significance (*p < 0.05, **p < 0.01, ***p < 0.001) were used, and p-value <0.05 was considered to be significant.

## Results

### Neddylation Pathway Overexpression Correlates With Global NEDD8 Levels and Predicts Poor Survival in Pancreatic Cancer

**Figure 1:**
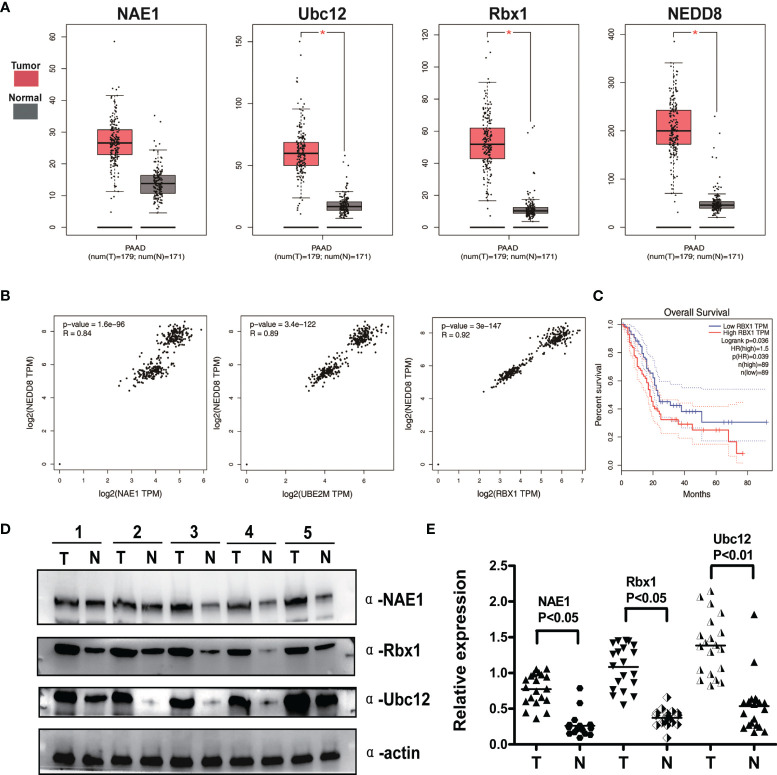
Neddylation pathway overexpression correlates with global NEDD8 levels and predicts poor survival in pancreatic cancer. **(A)** Bioinformatics analysis of The Cancer Genome Atlas (TCGA) RNA-Seq database in Gene Expression Profiling Interactive Analysis (GEPIA). Gene expression levels of NAE1, Ubc12, Rbx1 and NEDD8 were higher in pancreatic cancer tissues (n = 179) than adjacent normal tissues (n = 171), *p < 0.05. **(B)** Correlation between neddylation enzymes and global protein neddylation was analyzed in pancreatic cancer (Pearson’s chi-square test, TCGA data). **(C)** High expression of Rbx1 indicated a poor prognosis. Kaplan–Meier curves for overall survival rate of patients with pancreatic cancer according to the mRNA expression of Rbx1 with median gene expression level as the cutoff value (p < 0.05, log-rank test). **(D)** Immunoblotting (IB) analysis to determine the expression of NAE1, Rbx1, and Ubc12 in pancreatic adenocarcinomas tissues and adjacent normal tissues. Representative results of 5 out of 20 pairs of tissues are shown. T, tumor tissues; N, adjacent pancreatic tissues. **(E)** Quantification of protein expression in pancreatic tumor tissues compared with adjacent lung tissues (n = 20).

To assess the expression status of the neddylation pathway in pancreatic cancer, TCGA RNA-Seq dataset was performed to analyze the expression levels of NEDD8-activating enzyme E1 (NAE1) and NEDD8-conjugating enzyme E2 (Ubc12), E3 ubiquitin ligase Skp1/Cullin/F-box (Rbx1), NEDD8 in tumor vs. normal comparison, and patient overall survival. As shown in **Figure 1A**, Ubc12, Rbx1, and NEDD8 mRNA expression in pancreatic cancer tissues was notably higher than that in normal pancreatic tissues (p < 0.05). Moreover, correlation analysis revealed that the mRNA levels of NEDD8 and NAE1, Ubc12, and Rbx1 had a statistically significant association with pancreatic cancer (**Figure 1B**). Further Kaplan–Meier analysis indicated that patients with high mRNA levels of Rbx1 had worse overall survival than those with low expression in pancreatic cancer (**Figure 1C**). No significant correlation between the mRNA levels of NAE1, Ubc12, NEDD8, and the overall survival of pancreatic cancer patients was observed (data not shown). To confirm bioinformatics analysis results from TCGA and GEPIA databases, IB of 20 pairs of clinical pancreatic cancer tissues and adjacent normal tissues was performed to measure the protein expression of NAE1, Ubc12, and Rbx1. Consistent with the upregulation of mRNA expression levels, the protein expression levels of Ubc12 and Rbx1 were higher in pancreatic cancer tissues than in adjacent normal tissues (**Figures 1D, E**). NAE1 was also overexpressed in pancreatic tumor tissues vs. normal tissues (**Figures 1D, E**). The data collectively showed an overactivated status of the neddylation pathway in pancreatic cancer.

### Pevonedistat Impeded the Malignant Phenotypes of Pancreatic Cancer Cells *In Vitro*

**Figure 2:**
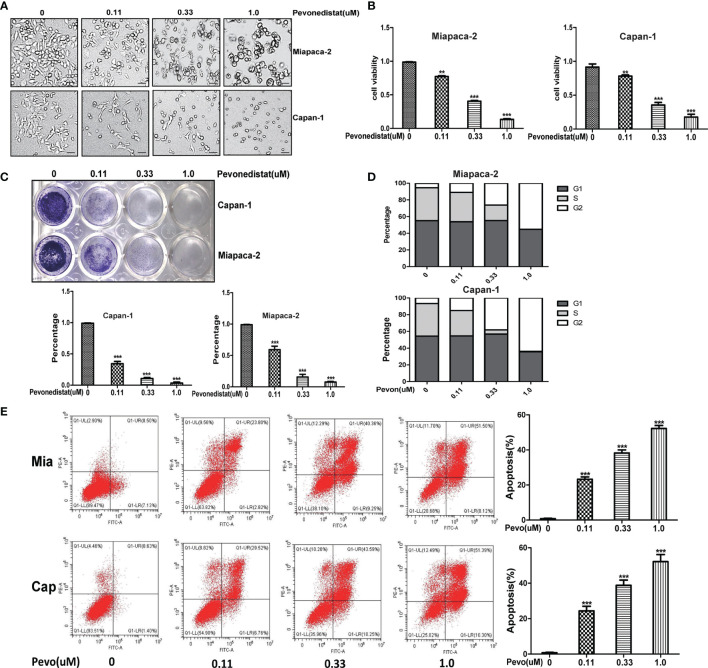
Pevonedistat impeded the malignant phenotypes of pancreatic cancer cells *in vitro*. **(A)** Growth suppression of pancreatic cancer cells by pevonedistat. Miapaca-2 and Capan-1 cells were treated with different doses of pevonedistat for 72 h and subjected to the micrograph. Scale bar = 200 μm. **(B)** Pevonedistat inhibited pancreatic cancer cell proliferation. A total of 2,500 Miapaca-2 and Capan-1 cells were seeded in 96-well plates in triplicates, treated with different doses of pevonedistat for 72 h, and then subjected for Cell Counting Kit-8 (CCK8) assay (mean ± SD, **p < 0.01, ***p < 0.001, n = 3). **(C)** Pevonedistat suppressed colony formation in pancreatic cancer cells. Miapaca-2 and Capan-1 cells were seeded into 12-well plates with 100 cells per well, cultured overnight, and then treated with indicated concentrations of pevonedistat for 10 days, followed by crystal violet staining and colony counting (mean ± SD, ***p < 0.001, n = 3). **(D)** Pevonedistat induced G2-M cell cycle defects. Miapaca-2 and Capan-1 cells seeded into 60-mm dishes were treated with pevonedistat at 0.11, 0.33, and 1.0 μM vs. dimethyl sulfoxide (DMSO) for 48 h, followed by propidium iodide (PI) staining and fluorescence-activated cell sorting (FACS) analysis for cell cycle profile. **(E)** Pevonedistat induced apoptosis in a dose-dependent manner. Miapaca-2 and Capan-1 cells, treated with pevonedistat at 0.11, 0.33, and 1.0 μM vs. DMSO for 72 h, were subjected to Annexin V-FITC/PI double-staining analysis. (mean ± SD, ***p < 0.001, n = 3). These data were representative results of three independent experiments with similar trends. Data represent means, and error bars are SD. Two-sided t-test.

Having shown the upregulated profiles of neddylation pathway members in pancreatic cancer, we next evaluated the effects of downregulation of the neddylation pathway on the malignant phenotypes of Miapaca-2 and Capan-1 cells treated with pevonedistat. The results of a Cell Counting Kit-8 assay clearly showed that pevonedistat significantly inhibited the cell growth phenotype (**Figure 2A**) and cell proliferation in a dose-dependent manner (**Figure 2B**). Pevonedistat also exhibited a strong suppressive effect on the clonogenic survival of these two pancreatic cancer cell lines (**Figure 2C**). According to FACS analysis, pevonedistat alone remarkably induced G2 phase arrest and apoptosis of Miapaca-2 and Capan-1 cells (**Figures 2D, E**). Altogether, these findings suggested that pevonedistat-induced inactivation of the neddylation pathway had a significantly effective anti-pancreatic cancer function.

### Depletion of the Neddylation Pathway Suppressed the Malignant Phenotypes of Pancreatic Cancer Cells *In Vitro*

**Figure 3:**
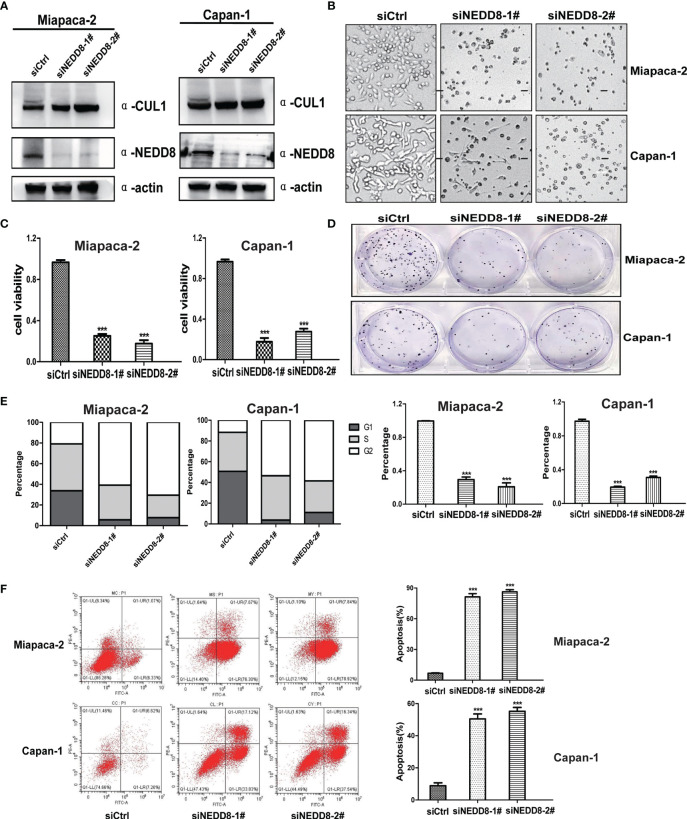
Depletion of the neddylation pathway suppressed the malignant phenotypes of pancreatic cancer cells *in vitro*. **(A)** siNEDD8 impaired Cullin neddylation. Miapaca-2 and Capan-1 cells were transfected with siControl and two siNEDD8 for 72 h and subjected to immunoblotting using antibodies against Cullin 1 and NEDD8 with actin as a loading control. **(B)** Growth suppression of pancreatic cancer cells by siNEDD8. Miapaca-2 and Capan-1 cells were transfected with siControl, siNEDD8-1#, and siNEDD8-2# for 72 h and subjected to the micrograph. Scale bar = 200 μm. **(C)** siNEDD8 inhibited pancreatic cancer cell proliferation. Miapaca-2 and Capan-1 cells were transfected by siControl, siNEDD8-1#, and siNEDD8-2#. After 24 h, 2,500 cells were seeded in 96-well plates in triplicates and then subjected for Cell Counting Kit-8 (CCK8) assay (mean ± SD, ***p < 0.001, n = 3). **(D)** siNEDD8 suppressed colony formation in pancreatic cancer cells. Miapaca-2 and Capan-1 cells were transfected by siControl, siNEDD8-1#, and siNEDD8-2#. After 24 h, 150 cells were seeded into 6-well plates for 10 days, followed by crystal violet staining and colony counting (mean ± SD, ***p < 0.001, n = 3). **(E)** siNEDD8 induced G2-M cell cycle defects. Miapaca-2 and Capan-1 cells were transfected with siControl, siNEDD8-1#, and siNEDD8-2# for 72 h, followed by propidium iodide (PI) staining and fluorescence-activated cell sorting (FACS) analysis for cell cycle profile. **(F)** siNEDD8 induced apoptosis in a dose-dependent manner. Miapaca-2 and Capan-1 cells were transfected with siControl, siNEDD8-1#, and siNEDD8-2# for 72 h and subjected to Annexin V-FITC/PI double-staining analysis. (mean ± SD, ***p < 0.001, n = 3). These data were representative results of three independent experiments with similar trends. Data represent means, and error bars are SD. Two-sided t-test.

To validate the direct link between pevonedistat and the neddylation pathway, we then assessed the effects of depletion of the neddylation pathway on the malignant phenotypes of Miapaca-2 and Capan-1 cells *via* knockdown of NEDD8. Consistent with pevonedistat, siNEDD8 markedly impaired Cullin neddylation compared with siControl in Miapaca-2 and Capan-1 cells (**Figure 3A**). Similarly, siNEDD8 largely suppressed pancreatic cancer cell growth and cell proliferation *in vitro* compared with siControl (**Figures 3B, C**). In the colony formation assay, NEDD8 knockdown showed an apparent suppressive effect on these two pancreatic cancer cell lines (**Figure 3D**). In FACS analysis, siNEDD8 led to cell cycle G2 phase arrest and apoptosis of Miapaca-2 and Capan-1 cells compared with the siControl (**Figures 3E, F**). Thus, these phenotype results revealed that depletion of the neddylation pathway also exhibited a significant anti-pancreatic cancer effect consistent with pevonedistat.

### Inactivation of the Neddylation Pathway Induced the Accumulation of Client Proteins Wee1, p27, and p21

**Figure 4:**
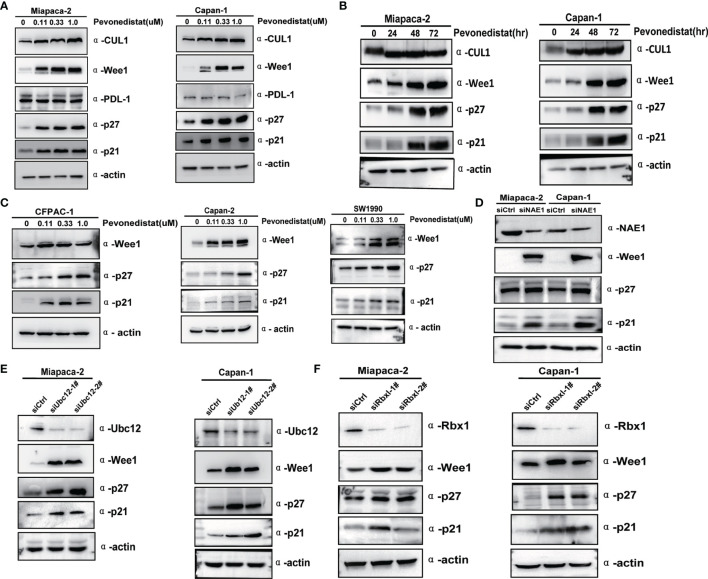
Inactivation of the neddylation pathway induced the accumulation of client proteins Wee1, p27, and p21. **(A)** Pevonedistat induced accumulation of Wee1, p27, and p21 in a dose-dependent manner. Forty-eight hours after Miapaca-2 and Capan-1 cells were treated with pevonedistat at increasing concentrations (0.1, 0.3, and 1.0 μM) vs. dimethyl sulfoxide (DMSO), cells were subjected to immunoblotting using antibodies against Cullin 1, Wee1, p27, and p21 with actin as a loading control. **(B)** Pevonedistat induced accumulation of Wee1, p27, and p21 in a time-dependent manner. Miapaca-2 and Capan-1 cells were treated with 1.0 μM of pevonedistat at increasing time points and then subjected to immunoblotting using antibodies against Cullin 1, Wee1, p27, and p21 with actin as a loading control. **(C)** Pevonedistat induced accumulation of Wee1, p27, and p21 in other pancreatic cancer cells. Forty-eight hours after CFPAC-1, Capan-2 and SW1990 cells were treated with pevonedistat at increasing concentrations (0.1, 0.3, and 1.0 μM) vs. DMSO, and cells were subjected to immunoblotting using antibodies against Cullin 1, Wee1, p27, and p21 with actin as a loading control. **(D)** siRNA knockdown NAE1 caused upregulation of Wee1, p27, and p21. Miapaca-2 and Capan-1 cells were transfected by siNAE1 and siCtrl for 72 h and then subjected to immunoblotting using antibodies against NAE1, Wee1, p27, and p21 with actin as a loading control. **(E)** siRNA knockdown Ubc12 led to upregulation of Wee1, p27, and p21. Miapaca-2 and Capan-1 cells were transfected by siUbc12 and siCtrl for 72 h and then subjected to immunoblotting using antibodies against Ubc12, Wee1, p27, and p21 with actin as a loading control. **(F)** siRNA knockdown Rbx1 resulted in upregulation of Wee1, p27, and p21. Miapaca-2 and Capan-1 cells were transfected by siRbx1 and siCtrl for 72 h and then subjected to immunoblotting using antibodies against Rbx1, Wee1, p27, and p21 with actin as a loading control. These data were representative of three independent experiments.

To elucidate the underlying molecular mechanisms of pevonedistat-mediated suppressive effects on pancreatic cancer cells, three predominantly restricted G_2_/M phase transition agents, Wee1, p27, and p21, were measured after treatment with pevonedistat. As shown in **Figures 4A, B**, with increasing concentration and time, pevonedistat apparently diminished basal neddylated Cullin 1 in Miapaca-2 and Capan-1 cells and strongly increased the expression of Wee1, p27, and p21 but not PDL-1 in a time- and dose-dependent manner, supporting our current phenotype analysis results. In addition, three other pancreatic cancer cells treated with pevonedistat also showed similar upregulation of Wee1, p27, and p21 (**Figure 4C**). To exclude the possible nonspecific effect of pharmaceutical treatment, siRNA knockdown of the neddylation pathway was then carried out. Accordingly, we found that siNAE1, siUbc12, and siRbx1 treatment led to the significant accumulation of Wee1, p27, and p21 (**Figures 4D**–**F**).

### Pevonedistat Prolonged the Half-Lives of Wee1, p27, and p21 and Reduced Their Ubiquitination Levels

**Figure 5:**
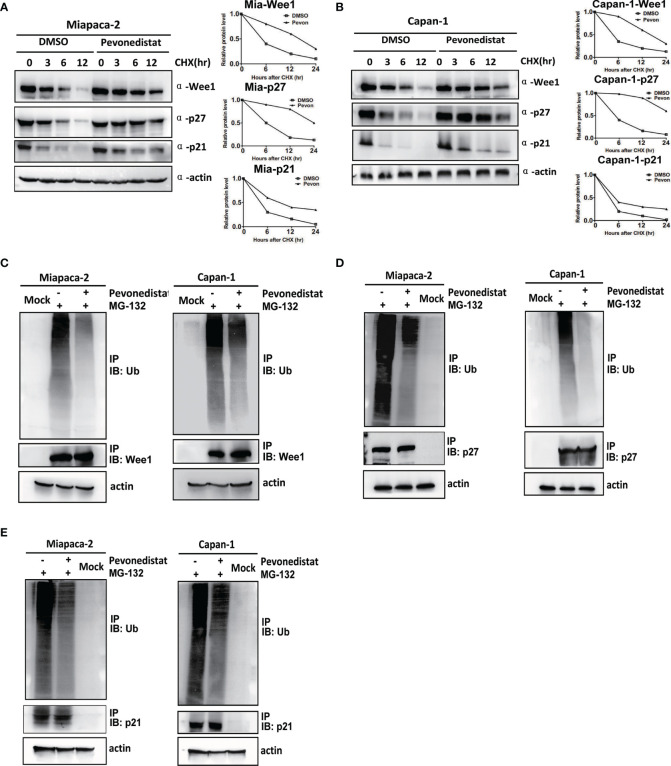
Pevonedistat reduced the ubiquitination levels of Wee1, p27, and p21, and prolonged their half-lives in pancreatic cancer cells. **(A)** Pevonedistat extended the half-lives of Wee1, p27, and p21 in Miapaca-2 cells. Cells were pretreated with pevonedistat for 24 h; then further treated by 1.0 μM of pevonedistat vs. dimethyl sulfoxide (DMSO) in combination with 50 μg/ml CHX for 0, 3, 6, and 12 h; and subjected to immunoblotting using antibodies against Wee1, p27, and p21 with actin as a loading control. The protein level was quantified by densitometry. Two-sided t-test. **(B)** Pevonedistat prolonged the half-lives of Wee1, p27, and p21 in Capan-1 cells. Cells were pretreated with pevonedistat for 24 h; then further treated by 1.0 μM of pevonedistat vs. dimethyl sulfoxide (DMSO) in combination with 50 μg/ml CHX for 0, 3, 6, and 12 h; and subjected to immunoblotting using antibodies against Wee1, p27, and p21 with actin as a loading control. The protein level was quantified by densitometry. Two-sided t-test. **(C)** Pevonedistat decreased the ubiquitination level of Wee1. Miapaca-2 and Capan-1 cells were treated with 1.0 μM of pevonedistat vs. DMSO for 24 h, then combined with 5 μM of MG-132 for more than 24 h, and subjected to immunoprecipitation using anti-Wee1 or rabbit IgG control antibody and immunoblotting using anti-ubiquitin antibody with actin as a loading control. **(D)** Pevonedistat reduced the ubiquitination level of p27. Miapaca-2 and Capan-1 cells were treated with 1.0 μM of pevonedistat vs. DMSO for 24 h, then combined with 5 μM of MG-132 for more than 24 h, and subjected to immunoprecipitation using anti-p27 antibody or rabbit IgG control antibody and immunoblotting using anti-ubiquitin antibody with actin as a loading control. **(E)** Pevonedistat attenuated the ubiquitination level of p21. Miapaca-2 and Capan-1 cells were treated with 1.0 μM of pevonedistat vs. DMSO for 24 h, then combined with 5 μM of MG-132 for more than 24 h, and subjected to immunoprecipitation using anti-p21 antibody or rabbit IgG control antibody and immunoblotting using anti-ubiquitin antibody with actin as a loading control. These data were representative of three independent experiments. Data represent means, and error bars are SD. Two-sided t-test.

Since Wee1, p27, and p21 are substrates of neddylation-CRL E3 ligases, we hypothesized that pevonedistat regulated their expression through protein post-translational modification. To verify this hypothesis, the ubiquitination levels and half-lives of Wee1, p27, and p21 in pevonedistat-treated Miapaca-2 and Capan-1 cells were determined upon treatment with the proteasomal inhibitor MG-132 and the protein synthesis inhibitor CHX, respectively. As shown in **Figures 5A, B**, Wee1, p27, and p21 were degraded gradually with CHX, whereas pevonedistat remarkably extended their half-lives during the process. Furthermore, MG-132 increased the endogenous ubiquitination of Wee1, p27, and p21, while pevonedistat treatment greatly decreased their ubiquitination levels compared with the control (**Figures 5C**–**E**). Taken together, the results indicated that pevonedistat significantly delayed the degradation of Wee1, p27, and p21 by inhibiting their ubiquitination levels.

### Pevonedistat Suppressed the Growth of Pancreatic Cancer *In Vivo*

**Figure 6:**
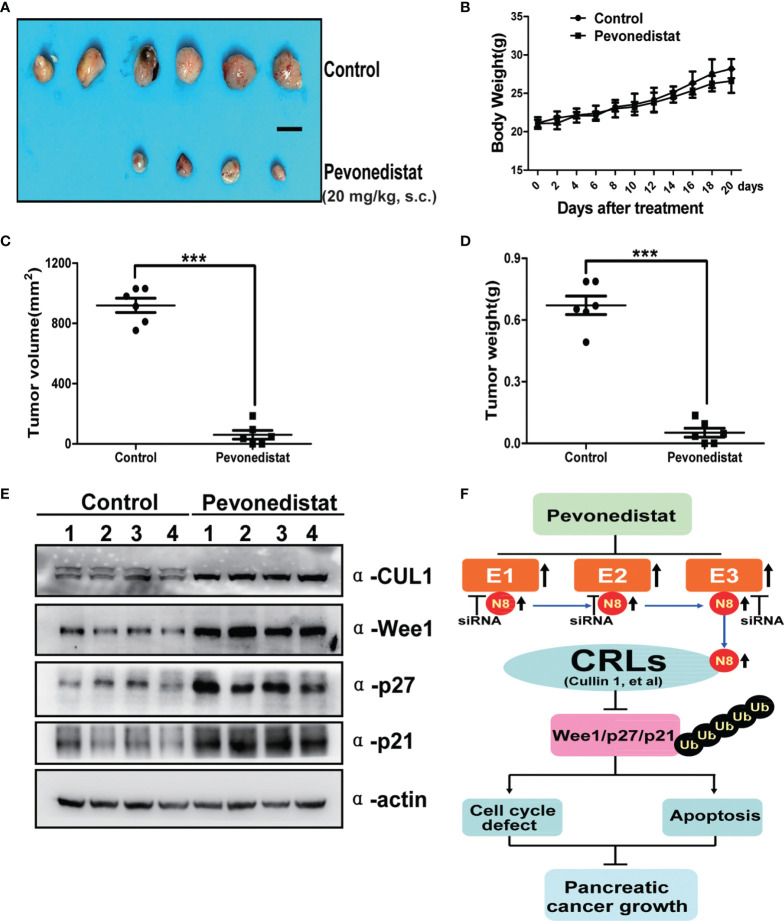
Pevonedistat suppressed the growth of pancreatic cancer *in vivo*. **(A–E)** Capan-1 cells measuring 1 × 10^7^ were injected subcutaneously and subjected to tumor growth analysis. Mice were sacrificed at the end of pevonedistat (20 mg/kg, s.c.) vs. control treatment (n = 6). **(A)** Tumor tissues of mice were collected and photographed (scale bar = 1 cm). **(B)** Mice were weighed during pevonedistat vs. control treatment schedule. **(C)** Tumor size was determined by caliper measurement (***p < 0.05). **(D)** Tumor was weighed on the sacrificed day (***p < 0.05). **(E)** Proteins extracted from tumor tissues and subjected to immunoblotting analysis against Cullin 1, Wee1, p27, and p21 with actin as a loading control. Data represent means, and error bars are SD. Two-sided t-test. **(F)** A working model of pevonedistat targeting neddylation pathway in pancreatic cancer. N8, NEDD8; CRLs, Cullin-RING E3 ubiquitin ligases.

After revealing the underlying anti-pancreatic cancer mechanism of pevonedistat, we further evaluated its cancer-inhibitory ability to target the hyperexpressed neddylation pathway in xenograft models *in vivo*. In the subcutaneously implanted tumor model, we found that pevonedistat significantly inhibited tumor formation and growth (**Figure 6A**) but not body weight gain (**Figure 6B**) in nude mice compared with the control groups, as analyzed by tumor size (**Figure 6C**) and tumor weight (**Figure 6D**). Next, mechanistic studies demonstrated that pevonedistat treatment impeded Cullin neddylation and resulted in obvious accumulation of Wee1, p27, and p21 (**Figure 6E**). Collectively, the results showed that pevonedistat inactivated the neddylation pathway and significantly suppressed the tumorigenesis and growth of pancreatic cancer *in vivo*.

## Discussion

The use of pevonedistat, a first-in-class anticancer agent that specifically targets overactivation of the neddylation pathway, has been defined as a potent anticancer strategy for multiple human cancers (18–20, 35). In our current study, we showed that the neddylation pathway was overexpressed in pancreatic cancer and that pevonedistat alone significantly inhibited pancreatic cancer malignant phenotypes, such as cell growth, cell proliferation, cell cycle, and tumor formation in nude mice. Mechanistically, the anticancer effects of pevonedistat on pancreatic cancer cells caused the accumulation of the cell cycle-related regulators Wee1, p27, and p21 and finally induced axis-mediated cell cycle arrest and apoptosis (**Figure 6F**). Our report, together with previous studies revealing the preclinical and clinical effects of pevonedistat on pancreatic cancer therapy (25, 30–32), provides rational evidence for the application of neddylation inhibitors for the treatment of pancreatic cancer in clinical trials.

Due to the extremely poor prognosis and high mortality of pancreatic cancer patients (1, 36), pharmacologic treatment for pancreatic cancer has proven difficult to achieve favorable outputs (6), despite gemcitabine being used extensively as the first-line therapy for advanced pancreatic cancer (3). The major challenge of chemotherapy is intrinsic resistance for those receiving definitive drug treatments. Thus, the combination of small molecule therapies, particularly targeted agents, such as pevonedistat, will effectively treat and manage pancreatic patients. Previous studies (25, 30–32) showed pevonedistat as a chemo- or radioadjuvant to improve the sensitiveness and efficacy of primary conventional treatment with a similar working model. Radiation or drug therapy always plays a killing role in cancer by inducing DNA damage and cell cycle defects. Pevonedistat synergistically enhanced these processes and ultimately led to apoptosis and cell death. CDT1 and Wee1, two key regulators of DDR and the cell cycle, and the best-known substrates of SCF E3 ubiquitin ligase were observed to accumulate and are involved in radiosensitization (25). A later study found that the proapoptotic protein NOXA and RAS signal inhibitor ERBIN were upregulated during the combination of gemcitabine and pevonedistat (31). Likewise, another study reported that p27 and p21, two cell division protein kinase (CDK) inhibitors, were obviously increased in the presence of pevonedistat and cisplatin in pancreatic cancer (32). These studies support our current finding that pevonedistat displays high efficacy in pancreatic cancer therapy. No study to date has specifically evaluated the monotherapy effect and demonstrated a more detailed intrinsic mechanism of pevonedistat targeting the neddylation pathway in pancreatic cancer.

Based on the mechanisms by which pevonedistat induced intrinsic cell growth suppression and apoptosis in pancreatic cancer cells, we highlighted the important roles of Wee1, p27, and p21 in this process. Li et al. (19) recently found that in lung cancer cells, pevonedistat-induced cell senescence was dependent on p27 and p21, and pevonedistat apparently increased the expression of p27 and p21. Wei et al. (25) showed that siRNA knockdown Wee1 partially rescued G2/M phase cell cycle arrest. In contrast, Soucy et al. (9) and Milhollen et al. (9) reported that pevonedistat could induce S phase cell cycle defects in colon cancer and breast cancer cells, and CDT1 knockdown in part retrieved cell rereplication upon pevonedistat treatment. In this study, we observed that neddylation inhibition by pevonedistat contributed to G2 phase defects and subsequent cell apoptosis in pancreatic cancer cells by suppressing the ubiquitination and degradation of Wee1, p27, and p21 and inducing their accumulation. The reasons for the heterogeneous results might be elicited by different cell types, diverse genetic backgrounds, heterogeneity of patients, and technical variability. Further elucidation of the intrinsic mechanisms of the cell line-dependent cell cycle and apoptosis regulation *via* Wee1, p27, and p21 with pevonedistat is warranted.

There are a few strengths of our study. The underlying mechanisms of neddylation pathway hyperexpression in pancreatic cancer have not been completely elucidated. How neddylation pathway members are activated in pancreatic cancer at the transcriptional level is unclear. For pancreatic cancer with a distinct cell context, for example, driven by KRAS or CDK, pevonedistat exhibits a similar anticancer effect and mechanism. The statistical evaluation of the clinical characteristics of pancreatic cancer patients was not included in this study. Further investigations are warranted to be performed.

## Conclusions

In summary, pancreatic cancer is the fourth most common malignant tumor worldwide. Personalized and novel therapeutic strategies for this heterogeneous disease are urgently needed. Our study showed that an aberrant hyperexpressed neddylation pathway was linked to pancreatic cancer development and progression. We further revealed the intrinsic mechanisms of pevonedistat targeting overactivation of the neddylation pathway and inducing G2 phase cell cycle defects and apoptosis in pancreatic cancer cells, which provide a scientific rationale for further clinical assessment of pevonedistat for the management of pancreatic cancer.

## Data Availability Statement

The original contributions presented in the study are included in the article/supplementary material. Further inquiries can be directed to the corresponding authors.

## Ethics Statement

The animal study was reviewed and approved by the Institutional Animal Care and Research Advisory Committee of Fudan University Shanghai Cancer Center.

## Author Contributions

This study was conceived by JX, WX, WL, XX, SJ, XX, and XY. JX, ZL, XX, WL, and WX designed the study. JX and ZL performed the experiments. JX, ZL, QZ, ZY, GF, HG, SJ, XX, XY, WL, and WX analyzed and interpreted the data. ZL, QZ, ZY, GF, HG, SJ, XX, XY, WL, and WX reviewed the manuscript. JX wrote the paper with comments from all authors. All authors read and approved the final manuscript.

## Funding

This work was jointly supported by the National Natural Science Foundation of China (81972250, 81871950, and 81972725), Scientific Innovation Project of Shanghai Education Committee (2019-01-07-00-07-E00057), Clinical and Scientific Innovation Project of Shanghai Hospital Development Center (SHDC12018109), Clinical Research Plan of Shanghai Hospital Development Center (SHDC2020CR1006A), and Shanghai Municipal Science and Technology Commission (19QA1402100).

## Conflict of Interest

The authors declare that the research was conducted in the absence of any commercial or financial relationships that could be construed as a potential conflict of interest.

## Publisher’s Note

All claims expressed in this article are solely those of the authors and do not necessarily represent those of their affiliated organizations, or those of the publisher, the editors and the reviewers. Any product that may be evaluated in this article, or claim that may be made by its manufacturer, is not guaranteed or endorsed by the publisher.

## Abbreviations

PAAD, pancreatic adenocarcinoma; NEDD8, neural precursor cell expressed developmentally downregulated 8; NAE1, NEDD8-activating enzyme E1; CRL, Cullin-RING E3 ligase; ROS, reactive oxygen species; DDR, DNA damage response; CHX, cycloheximide; IB, immunoblotting; IP, immunoprecipitation; PI, propidium iodide; FACS, fluorescence-activated cell sorting; HPBCD, 2-hydroxypropyl-β-cyclodextrin; SPSS, Statistical Package for the Social Sciences software.
